# Addictive behaviors, cardiovascular and metabolic comorbidities in North African psoriatic patients: case-control study

**DOI:** 10.11604/pamj.2019.34.205.12883

**Published:** 2019-12-19

**Authors:** Amina Aounallah, Fatim-Zahra Mernissi, Boumediene Dahmani, Iheb Bougmiza, Sahel Houria, Bacar Bouadjar, Ismail Benkaidali, Aomar Ammar-Khodja, Amina Serradj, Abdelhamid Titi, Badreddine Hassam, Hakima Benchikhi, Said Amal, Raouf Dhaoui, Hamida Turki, Mokhtar, Nejib Doss, Mohamed Denguezli

**Affiliations:** 1 Farhat Hached Hospital, Dermatology Department, Sousse, Tunisia; 2 Faculty of Medicine, Dermatology Department, Fes, Morocco; 3 Centre Hospitalo-Universitaire Dr Tidjani Damerdji, Département de Dermatologie, Tlemcen, Algeria; 4 Université de Sousse, Faculté de Médecine de Sousse, Département d’Epidémiologie, Sousse, Tunisia; 5 CHU Bab El Oued, University of Algiers, Dermatology Department, Alger, Algeria; 6 CHU Mustapha, Algiers, Algeria; 7 CHU Oran, University of Oran, Dermatology Department, Algeria; 8 CHU Annaba, University of Annaba, Dermatology Department, Annaba Algeria; 9 University Hospital Casablanca, Morocco; 10 Faculty of Medicine, Dermatology Department, Marrakech, Morocco; 11 Military Hospital of Instruction of Tunis, Tunisia; 12 CHU Hedi Chaker, Dermatology Department, Sfax, Tunisia; 13 Hopital Habib Thameur, Département de Dermatologie, Tunis, Tunisie

**Keywords:** Psoriasis, comorbidies, North Africa

## Abstract

We propose to study the epidemiological aspects of North African psoriasis and determine the cardiovascular comorbidities and addictive behaviors associated with psoriasis. This is a North African case-control study which was conducted over a five year period (October 2008 through August 2013), involving 671 psoriatic patients and 1,242 controls identified in various Algerian, Tunisian and Moroccan university hospitals. For each patient, epidemiological characteristic, addictive behaviors, and cardiovascular pathologies associated with psoriasis were noted. Six hundred and seventy one psoriasis patients and 1,242 controls were included in this study. The average age was 47.24 years and the M/F sex-ratio was 1.11 (354 men and 317 women). Statistical analysis showed that psoriasis patients were more likely to develop addictive behaviors than controls (smoking p<10-5 and alcohol consumption: p < 10-5), together with dyslipidemia (30.1% of patients p < 10-5), obesity (23.8% of patients p < 10-4), hypertension (22.3% of patients p < 10-5), diabetes (21.7% of occurrences p < 10-5) and metabolic syndrome (37.4% of patients p<10-5). The relative risk for developing psoriasis was 1.9 in hypertensive patients, 1.7 in diabetic patients, 3.9 in dyslipidemic patients, 1.8 in obese patients, 2.6 in those with metabolic syndrome, 2.1 in smokers and 2.8 in alcoholics. Our work confirms the high incidence of addictive behaviors and of cardiovascular and metabolic comorbidities during the North-African psoriasis, hence the need for a multidisciplinary comprehensive care based on a guideline suited to the characteristics of North-African psoriatic patients.

## Introduction

Psoriasis is a chronic inflammatory and visible skin disease occurring in a particular genetic ground [1]. It is one of the most common autoimmune diseases, affecting 2-3% of the world's population [2]. Its incidence in the Maghreb is estimated to be 12.08 per 1,000 inhabitants [3]. Psoriasis is currently considered as an immune-mediated inflammatory disease likely to reduce patients 'quality of life and result in considerable physical disability. It is associated with significant psychological burden [4,5]. While in most cases psoriasis is benign, in 10% of cases it can be serious (because of its spread on the body or its complications) [6]. Psoriasis appears to reduce lifespan by 3.5-10 years, with early disease onset reducing life expectancy by up to 20 years [7]. According to literature data, several studies have shown a clear association between psoriasis and other diseases, in particular among young patients with severe psoriasis. Associated pathologies are essentially cardiovascular, metabolic, inflammatory and neuropsychological disorders [1,7,8]. Several non-recurrent publications from Maghreb countries have focused on the study of these comorbidities [3,9]. However, no large-scale work on cardiovascular comorbidities including Tunisian, Algerian and Moroccan patients in the same series had been published to date. Purpose: are the cardiovascular comorbidities and addictive behaviors associated with psoriasis in North African population?

## Methods

This was a North African matched case-control study involving 671 patients with psoriasis and 1,242 controls identified in various Algerian (Psoriatic: 368, Control: 736), Tunisian (Psoriatic: 153, Control: 206) and Moroccan (Psoriatic: 150, Control: 300) university hospitals, focusing on a population aged over 18. Controls were also attending various dermatology departments for skin diseases other than psoriasis. They were matched to the cases on age and gender. Data was prospectively collected over a 5 year period (October 2008-August 2013). Psoriasis diagnosis based on the anamnestic and clinical data or confirmed by histology if in doubt. All clinical forms of psoriasis were included and no exclusion criteria were retained for enrollment. Controls were excluded from the study if they had been treated with local or systemic corticosteroids. For each patient, we had filled in an information sheet including the epidemiological characteristics (age, gender), assessment of addictive behaviors (smoking, alcoholism) and whether or not psoriasis was associated with cardiovascular diseases (hypertension, diabetes, dyslipidemia, obesity and metabolic syndrome). Blood pressure was taken as the average of the two measurements after the patient was allowed to rest for 15 minutes. Hypertension is defined as a blood pressure greater than or equal to 140/90 mm Hg. The following blood tests were conducted on patients after 12 hours of fasting (glucose, triglyceride, total cholesterol, high density lipoprotein cholesterol (HDL cholesterol) and low density lipoprotein cholesterol (LDL cholesterol). Those with a fasting blood glucose level equal to or greater than 1.26 g/l were considered diabetic. Dyslipidemia was retained in patients with elevated levels of one or more serum lipids (triglycerides, total cholesterol, LDL cholesterol) and/or reduced HDL cholesterol levels. Dyslipidemia is defined as triglyceride level ≥ 1.7 mmol/l (1.5 g/l) and/or total cholesterol level ≥ 6.45 mmol/l (2.5 g/l) and/or LDL cholesterol ≥ 4.13 mmol/l in the absence of cardiovascular risk factors or LDL cholesterol ≥ 3.35 mmol/l in the presence of cardiovascular risk factors. According to the National Cholesterol Education Program Adult Treatment Panel III (NCEP ATP III) definition, metabolic syndrome is present if three or more of the following five criteria are met: waist circumference ≥80cm for women and ≥94cm for men; hypertriglyceridemia ≥1.70 mmol/l (150 mg/dl); high-density lipoprotein (HDL) cholesterol level < 1.23mmol/l (40 mg/dl) for men < 1.29 mmol/l (50 mg/dl) for women; high blood pressure: systolic ≥ 130mm Hg or diastolic ≥ 85mm Hg; Hyperglycemia ≥ 6.1 mmol/l(110 mg/dl). Patients, receiving a hypolipemiant, antihypertensive or antidiabetic treatment at the time of examination, were considered carriers of the disease for which they were treated. Statistical analysis was performed using SPSS.

## Results

Six hundred and seventy one psoriatic patients and one thousand two hundred and forty-two control subjects were included in this study. The average age was 47.24 years and the M/F sex-ratio was 1.11 (354 men and 317 women). Research on addictive behaviors revealed that 30.2% of patients were smokers compared to 16.9% of control subjects (p <10-5) and that 8.1% of patients were alcoholics (versus 3% of controls, p <10-5). Analysis of cardiovascular risk factors showed that hypertension was found in 22.3% of patients (versus 13.1% of controls p <10-5), diabetes in 21.7% of cases (versus 13.8% p <10-5), dyslipidemia in 30.1% of patients (versus 9.9% of controls p <10-5), obesity in 23.8% of patients (versus 14.2% of controls p<10-4) and the metabolic syndrome in 37.4% of patients (versus 18.3% of controls p <10-5). The relative risk for developing psoriasis was 1.9 in hypertensive patients, 1.7 in diabetic patients, 3.9 in dyslipidemic patients, 1.8 in obese patients, 2.6 in those with metabolic syndrome, 2.1 in smokers and 2.8 in alcoholic patients (Table 1).

**Table 1:** Addictive behaviors, cardiovascular and metabolic comorbidities in North African psoriatic

Co-morbidities		Psoriatic patients N=671	Controls N=1241	P	OR	95% CI
Hypertension	Yes	150 (22.3)	163 (13.1)	<10^-5^	1.9	1.4 – 2.4
	No	521 (77.7)	1,078 (86.9)			
Diabetes	Yes	146 (21.7)	172 (13.8)	<10^-5^	1.7	1.3 – 2.2
	No	525 (78.2)	1,069 (86.2)			
Dyslipidemia	Yes	202 (30.1)	123 (9.9)	<10^-5^	3.9	3.0 – 5.0
	No	469 (69.9)	1118 (90.1)			
Obesity	Yes	160 (23.8)	177 (14.2)	<10^-4^	1.8	1.4 – 2.3
	No	511 (76.2)	1,064 (85.8)			
Smoking	Yes	203 (30.2)	210 (16.9)	<10^-5^	2.1	1.7 – 2.6
	No	468 (69.8)	1,031(83.1)			
Alcohol consumption	Yes	55 (8.1)	38 (3.0)	<10^-5^	2.8	1.8 – 4.3
	No	616 (91.9)	1,203 (97.0)			
Metabolic Sd	Yes	251 (37.4)	228 (18.3)	<10^-5^	2.6	2.1 – 3.2
	No	420 (66.6)	1,013 (81.7)			

## Discussion

Our work confirms the high incidence of addictive behaviors and cardiovascular and metabolic comorbidities during North-African psoriasis. Psoriasis is a common skin disease in the Maghreb that is often under-diagnosed [3]. It is a condition frequently associated with extracutaneous manifestations and is now considered a real systemic disease [1]. The chronic inflammatory nature of psoriasis, in particular the high levels of proinflammatory cytokines such as TNFα, as well as certain common genetic features predispose patients to develop addictive behaviors (smoking, alcohol consumption) and multiple co-morbidities [10,11]. Smoking is a risk factor for many chronic diseases, including psoriasis [10], especially in the pustular forms [12]. The pathophysiology of the link between smoking and psoriasis involves changes in neutrophil chemotaxis [13]. In fact, cigarette smoke induces overproduction of proinflammatory cytokines (TNFα, IL2, IL6, IL8) and chemokines whose role in exacerbating psoriasis is unanimously recognized [13]. On the other hand, the negative impact of the disease on the quality of life of psoriatic patients and their psychological state will promote active smoking and alcohol dependence [14]. Some have reported the potential impact of alcohol on the severity, clinical presentation as well as evolution and prognosis of psoriasis, concluding that alcohol may not only trigger but also worsen psoriasis [15]. Alcohol, through its metabolic and immunological effects, may be involved in new-onset and flare of psoriasis. It may stimulate keratinocyte proliferation and production of proinflammatory cytokines. It also causes vasodilation and increased vascular permeability, thereby promoting neutrophil migration and epidermal infiltration [15]. This overconsumption of alcohol may precede psoriasis or be secondary to the impact of psoriasis on patients' psycho-emotional state, in particular depression [16]. Research on addictive behaviors in our patients confirmed the above-mentioned findings, since 30.2% of patients were smokers versus 16.9% of controls (p < 10^-5^) and 8.1% of patients were alcoholics (versus 3% of controls, p < 10^-5^). Our results may underestimate actual alcohol use, since some patients may be reluctant to report their actual consumption, as has been observed in most epidemiological studies involving patients with psoriasis. Our results are different from other epidemiological studies, firstly because they come from other countries, primarily European, where social drinking habits are quite different from ours and also due to the lack of accuracy in our study with respect to the definition and quantification of alcohol consumption, which have not been assessed. Recent studies have shown that psoriasis was associated with increased obesity-related inflammatory markers, such as TNFα, interleukin 6 and interleukin 8 in adipose tissue. TNFα will lead to decreased adiponectin secretion (CK secreted by adipose tissue) [17,18].

This decrease in adiponectin will improve insulin resistance but also increase triglycerides, LDL-C cholesterol and decrease HDL-C. This hyperlipidemia induces obesity, thereby triggering proinflammatory cytokine production by adipocytes and macrophages and establishing a vicious circle [17,18]. Studies suggest that obesity may be a risk factor for the development of psoriasis [1] but other studies shown elevated body mass index in patients after the diagnosis of psoriasis, meaning that obesity appears to be a consequence, rather than a cause of psoriasis [19]. The prevalence of obesity in the psoriatic population appears to be twofold higher than in the general population (34% versus 18%, P <0.001) [8]. These same results have also been observed in our North African psoriatic patients. Previous studies have reported an increased prevalence of diabetes in patients with psoriasis [4]. This association is thought to be related to the chronic nature of inflammation in psoriasis, with high levels of TNFα, CRP, IL-6 [20]. Among adipocytokines, leptin level is elevated in psoriasis and diabetic patients while adiponectin level is decreased [20]. Hyperleptinemia in psoriasis may have a role in metabolic syndrome development [21]. Many studies suggest a strong link between the development of psoriasis and diabetes [19]. These results were observed during an Israeli retrospective study, which confirmed the increased risk for diabetes mellitus in patients with psoriasis (odd ratio = 1.3) on a large population of 46,095 psoriatic patients and 1,579,037 controls [22]. However, further scientific work found that there was no significant difference between psoriatic and control patients with respect to the prevalence of type II diabetes [23]. In most cases whether diabetes precedes or follows the onset of psoriasis is not known, but studies on the incidence of diabetes suggest that most patients develop diabetes after being diagnosed with psoriasis [24]. Similar genetic predisposition for insulin resistance may also explain the higher prevalence of diabetes among psoriasis patients [25,26]. Another possible explanation for the association between psoriasis and diabetes is the presence of chronic inflammation that occurs due to persistent secretion of TNF-α and other proinflammatory cytokines such as IL-1 and IL-6, which precipitates both psoriasis and diabetes [25,26]. These cytokines stimulate the hepatic production of acute phase proteins of inflammation such as CRP [25,26] which is elevated in minimal to severe psoriasis [27]. This chronic systemic inflammation induces endothelial dysfunction, altered glucose metabolism and insulin resistance that play a significant role in the development of diabetes and psoriasis [28]. In our North African patients, diabetes was found in 21.7% of cases (versus 13.8% p < 10-5). The association of psoriasis with dyslipidemia had been discussed for many years. Multiple studies have found psoriasis to be associated with dyslipidemia [23]. Most of these studies reported hypertriglyceridemia, high total cholesterol levels or very-low-density lipoprotein cholesterol (VLDLC) and low density lipoprotein (LDL), as well as lower levels of high density lipoprotein cholesterol (HDLC) [23]. The role of systemic therapies, in particular retinoids, was reported together with smoking and obesity [29]. However, the results are not consistent enough. In fact, a few studies reported no differences in lipid serum levels between psoriatic patients and healthy controls [30]. We do not know whether these dyslipidemias are primary events that may trigger psoriasis development or if they are secondary to many therapeutic, inflammatory or genetic factors [30].

We can assume that obesity (which is found in 23.8% of our psoriatic patients) may be the factor linking psoriasis to alterations in lipid metabolism. It has been demonstrated that adipocytes influence the lipid profile by increasing the levels of pro-inflammatory cytokines, in particular TNF-α and IL-6, which may affect the rate of free fatty acids, cholesterol and lipids [29]. However, more recent studies underscored that a particular lipid profile involving high cholesterol concentrations in VLDL fractions, independent of the following factors (age, gender, BMI, smoking status, blood pressure, physical activity and alcohol intake) is observed from the onset of psoriasis [29]. This independence suggests that the atherogenic dyslipoproteinemia may be genetic rather than acquired [29]. The association between psoriasis and high blood pressure is now known [24]. It could be attributed to angiotensin II, an angiotensin-converting enzyme degradation product that regulates vascular tone and stimulates the release of proinflammatory cytokines [24]. Our results confirm those in the literature since 22.3% of North African patients were found to have high blood pressure (versus 13.1% of controls p < 10-5). Many studies suggest that psoriasis is often associated with MS, to the extent that some suggested that psoriasis may be one of the components of the metabolic syndrome [2,22,30]. The pathophysiological links between psoriasis and MS are essentially based on the common proinflammatory mechanism of both pathologies resulting from TNF, IL6 and many adipokines, including leptin [2,22,30]. Our study found a significantly higher prevalence of MS among psoriatic patients compared to controls (37.4% versus 18.3% p<10-5). These results are not consistent with those from the Tunisian study of Mebazaa *et al* [11], where the prevalence of MS was found to be higher among psoriatic patients compared to controls, yet with no statistical difference (35.5% versus 30.8%, OR = 1.39, (95%CI : 0.88-2.18), p = 0.095). The prevalence of MS differs from one study to another due to the lack of homogeneity between studies, for a number of reasons: Studies come from different countries, which implies genetically and racially diverse populations with different life style patterns. These differences influence the prevalence of MS. Controls were included from different population samples. Psoriatic patients were selected from electronic medical record databases, or recruited from the dermatology consultations or groups of hospitalized psoriasis patients. Adjustment for age, gender, or place of residence has not systematically been made. Lifestyle-related factors, including smoking, alcohol consumption, physical activity and use of systemic therapies for psoriasis (cyclosporine, retinoids) were often not taken into account. The retrospective or prospective nature of enrollment and results' evaluation as well as the matching differed from one study to another. This considerable heterogeneity has resulted in non-comparable results and is a source of considerable variations in prevalence

## Conclusion

Our work confirms the high incidence of addictive behaviors and of cardiovascular and metabolic comorbidities during the North-African psoriasis, hence the need for a multidisciplinary comprehensive care based on a guideline suited to the characteristics of North-African psoriatic patients.

### What is known about this topic

- Psoriasis is now considered as a systemic disease;
- The association of psoriasis with hypertension, obesity, diabetes, metabolic syndrome and dyslipidemia is reported in the world;
- Psoriatic patients have more addictive behaviors than normal population.

### What this study adds

- It has allowed us to assess the prevalence cardiovascular comorbidities and addictive behaviors associated with psoriasis;
- Our study suggests that metabolic syndrome is present in our population as frequently as in developed countries and should be evaluated and treated.

## Competing interests

The authors declare no competing interests.
